# Revised Device Labeling for the Cepheid Xpert MTB/RIF Assay for Detecting *Mycobacterium tuberculosis*

**Published:** 2015-02-27

**Authors:** Division of Microbiology Devices, Office of In Vitro Diagnostics and Radiological Health, Center for Devices and Radiological Health, Food and Drug Administration

The Food and Drug Administration (FDA) has cleared the Xpert MTB/RIF Assay (Cepheid; Sunnyvale, California) with an expanded intended use that includes testing of either one or two sputum specimens as an alternative to examination of serial acid-fast stained sputum smears to aid in the decision of whether continued airborne infection isolation (AII) is warranted for patients with suspected pulmonary tuberculosis (*1*). This change reflects the outcome of a recent multicenter international study demonstrating that negative Xpert MTB/RIF Assay results from either one or two sputum specimens are highly predictive of the results of two or three negative acid-fast sputum smears.*

When compared with the results of two or three serial fluorescent-stained acid-fast sputum smears, a single Xpert MTB/RIF Assay result detected approximately 97% of patients who were acid-fast bacilli (AFB) smear–positive and culture-confirmed as infected with *Mycobacterium tuberculosis* complex (MTBC), and two serial Xpert MTB/RIF Assay results detected 100% of AFB smear–positive/MTBC culture-positive patients. In the setting of an overall prevalence of culture-confirmed pulmonary tuberculosis of 22.4% (14.2% [88 of 618] in the United States and 37.1% [127 of 342] outside the United States), a single negative Xpert MTB/RIF Assay result predicted the absence of AFB smear–positive pulmonary tuberculosis with a negative predictive value of 99.7% (99.6%% in the United States and 100% outside the United States); for two serial negative Xpert MTB/RIF Assay results, the negative predictive value was 100%. These findings confirm the results from earlier reports (*2,3*). In addition, one or two Xpert MTB/RIF Assay tests detected 55% and 69%, respectively, of sputum specimens that were AFB smear–negative but culture-positive for MTBC.

Updated labeling for the Xpert MTB/RIF Assay includes the recommendation that the decision whether to test one or two sputum specimens in determining the need for continued AII should be based on specific clinical circumstances and institutional guidelines. Clinical decisions regarding the need for continued AII should always occur in conjunction with other clinical and laboratory evaluations, and negative Xpert MTB/RIF Assay results should not be the sole basis for infection control practices. The revised label also includes information demonstrating that Xpert MTB/RIF Assay performance is similar in human immunodeficiency virus (HIV)-infected and HIV-uninfected adults, although HIV-infected adults with pulmonary tuberculosis might be more likely to be AFB smear negative at presentation. The Xpert MTB/RIF Assay should not be used for decisions regarding the need for continued AII if MTBC has been detected by the Xpert MTB/RIF Assay or by other methods.

Product labeling retains the recommendation that regardless of Xpert MTB/RIF Assay results, serial collection of sputum specimens for mycobacterial culture remains necessary because nucleic acid amplification testing does not detect all patients with pulmonary tuberculosis, and recovery of organisms for further characterization and drug-susceptibility testing is needed when MTBC is present. Concomitant acid-fast microscopy of serial sputum specimens is also needed when excluding nontuberculosis mycobacterial disease. Readers are encouraged to review the updated product labeling and the previous related *MMWR* report for additional information regarding the Xpert MTB/RIF Assay (*1,4*).

## Footnotes

* These results represent independent FDA analysis of results from study ACTG A5295/TBTC 34. Additional information is available from Luetkemeyer A, Firnhaber C, Kendall M, et al., on behalf of the ACTG A5295/TBTC 34 study teams. Xpert MTB/RIF versus AFB smear to determine respiratory isolation of U.S. TB suspects. Presented at the Conference on Retroviruses and Opportunistic Infections, February 23–26, 2015, Seattle, Washington.
